# Exploring the combat mindset approach during a major prison riot

**DOI:** 10.3389/fpsyg.2026.1738405

**Published:** 2026-04-02

**Authors:** Knut Mellingsæter Sørensen, Ole Boe, Per Eirik Lund

**Affiliations:** 1 Department of Correctional Studies, Section for Security, University College of Norwegian Correctional Service, Lillestrøm, Norway; 2 USN School of Business, Department of Business, Strategy and Political Sciences, University of South-Eastern Norway, Drammen, Norway; 3 University of South-Eastern Norway, Porsgrunn, Norway

**Keywords:** combat mindset, decision making, situational awareness, stress management, training methods

## Abstract

This autoethnographic study explores the mindset of prison officers in a prison riot in Oslo, Norway, addressing two research questions (RQs): RQ1: How does a prison officer’s experience during a riot align with the concept of a well-functioning combat mindset? RQ2: Is there a need for more stressful training scenarios for prison officers to foster this mindset? To explore our two RQs, we analyzed a prison officer’s autoethnographic account of a full-scale riot. Findings indicate that the officer’s experience of dealing with a situation with extreme stress supports the combat mindset concept, affirming the first research question. The study also concludes that extensive training is necessary to develop controlled aggression to the situation at hand, not resulting in serious injury. Training is crucial in order to manage high-stress situations effectively, supporting the second research question. This case study underscores the importance of a well-functioning combat mindset for security professionals, achieving balanced responses under extreme stress in order to deal with people who have the intention to harm others in frontline operations.

## 1 Introduction

Several security professionals face demanding work environments that can be a source of severe stress and, in some cases, life-threatening situations. Such professions are according to Reiner (1997, p. 997) practitioner policing, meaning ‘the whole craft of governing a social order’. First, multiple studies highlight the necessity for soldiers in the military to develop a well-functioning combat mindset to handle situations where their lives are at risk (Asken et al., 2010; Boe and Ingdahl, 2017; Siddle, 1995). While research on police officers has not been as extensively examined as that on military personnel, one study argues that police officers should undergo more training in developing a well-functioning combat mindset, particularly in light of the police response during the terror attacks in Norway at the government headquarters and on Utøya island, where 77 people were killed (Boe et al., 2020). These attacks remain the deadliest in Norway since World War II. The third security profession, the Norwegian Correctional Service (NCS), involves prison officers working directly with demanding inmates. Maximum-security prisons in Norway are structured with a primary focus on security, rehabilitation, and resocialization. Norway is globally recognized for its humane approach to punishment and incarceration, which is reflected in the organization of its maximum-security prisons (Pratt, 2008a, 2008b; Pratt and Eriksson, 2013; Smith and Ugelvik, 2017).

### 1.1 Is there a lack of focus on a well-functioning combat mindset in the Norwegian correctional service?

According to Norwegian Government White Paper, the NCS focus more on rehabilitation than security concerns (Justis-og beredskapsdepartementet, 2008). However, the NCS also faces severe situations where lives may be at risk. Sørensen (2023) describes violence, hostage situations, prison disturbances, and prison riots in Norway that pose life-threatening dangers to frontline prison officers. In international literature, several contributions have explored various elements of prison riots, providing insights into their causes and management strategies.

More recently, collective riots and disturbances have been reported in Semb prison (2019), Oslo prison (2020), and Ullersmo prison (2024) in Norway, all of which required police intervention to restore order (Sørensen, 2023). In Norway there is very little research on prison riots and disturbances. The international literature includes various contributions aimed at understanding different aspects of prison riots (Boin and Rattray, 2004; Boin and Van Duin, 1995; Player and Jenkins, 1993; Useem and Kimball, 1989; Useem and Piehl, 2006; Useem and Reisig, 1999).

### 1.2 Aim of the study and research questions

In this article we will give an autoethnographic description of a prison riot in Oslo prison in Norway in New Year’s Eve in the year 2000 by the third author. We thus aim at exploring this prison riot through two research questions: RQ1: How does a prison officer’s experience during a riot align with the concept of a well-functioning combat mindset? RQ2: Is there a need for more stressful training scenarios for prison officers to foster this mindset? To get further insight into this phenomenon of this article, the third author will give a unique insight into a Norwegian prison riot that took place in 2000. In line with Boe et al. (2020), we argue that a well-functioning combat mindset enhances an individual’s operational performance in unpredictable and threatening situations, improving both aggression, aggression control and the ability to neutralize dangerous threats, and that a well-functioning combat mindset is a necessity for prison officers during prison riots.

### 1.3 The Norwegian correctional service: security levels, disturbances and violence

Norway has a population of approximately 5.4 million (SSB, 2025). Norway operates a small prison system with around 56 facilities housing about 3,000 inmates (Kriminalomsorgen, 2021). The focus in maximum-security prisons in Norway is on rehabilitation and reintegration alongside incarceration, aiming to reduce recidivism through educational and vocational training (Eide and Westrheim, 2020; Fredwall, 2017; Pratt, 2008b, 2008a).

Scandinavian prisons, including Norway’s, are regarded as exceptional (“Nordic penal exceptionalism”) due to their unique prison model (Pratt, 2008a,b; Ugelvik and Dullum, 2012). Norwegian prisons are smaller and have a higher staff-to-inmate ratio than those in the US, UK, and Canada. Officers take on a more social role, reflecting Norway’s egalitarian and less authoritarian prison model (Pratt, 2008a,b; Ugelvik and Dullum, 2012).

Inmates are classified into three security levels, where high-security prisons featuring strict measures such as tall fences, constant surveillance, and limited overnight freedom. Prison officers in these facilities balance enforcement of rules and regulations with situational awareness to detect risks and maintain control (Midtlyng, 2022, 2024). Unwanted incidents occur in both high- and low-security prisons, but large-scale riots still remain relatively rare in Norway.

Due to a small prison population and relatively few serious incidents, Norway has not established a national response team. Instead, each prison handles incidents locally, with the option to request police assistance in serious cases, who possess more extensive powers than the prison authorities. In the Oslo Prison riot analyzed in this article, the incident commander had to rely on the officers immediately available at the prison when the disturbance began. Although the preferred approach was to wait to go in with the riot squad and enter the prison wing, the outbreak of fire forced immediate action. To prevent the fire from spreading and to protect inmates locked in their cells from smoke and heat, immediate action was required. Officers wore protective equipment, including helmets, batons, and padded suits. Norwegian prison officers are trained to handle riots through tactical and operational training during their two-year education at the University College of the Norwegian Correctional Service (UCNCS). Nevertheless, the role of a prison officer is marked by significant contrasts. Prison officers are trained as generalists, balancing roles that emphasize care and rehabilitation with the need to respond to serious prison riots. To understand how officers shift between tasks requiring aggression and those that do not, this article examines the concept of a well-functioning combat mindset and what this means for prison officers.

### 1.4 Components of a well-functioning combat mindset

One definition of the term combat mindset is the following: “Willingness and ability to continue the fight despite the high levels of mental and physical pain” (Boe, 2006, 2007). Handling individual and collective prison riots is highly demanding, requiring prison officers to adopt a well-functioning combat mindset. Krulak (1999) emphasizes that military operations demand adaptability across various tasks, a principle reflected in a well-functioning combat mindset comprising three key elements (Siddle, 1995). First, it involves courage, determination, mental stamina, and aggression, enabling officers to confront fear and perform under pressure (Yanilov and Boe, 2020). Second, it includes stress management techniques such as controlled breathing, mindfulness, and mental rehearsal to regulate emotions like fear, anger, and frustration (Yanilov and Boe, 2020). Lastly, focus, concentration, and self-control ensure disciplined responses and clarity in high-pressure situations (Yanilov and Boe, 2020).

A well-functioning combat mindset also includes technical, physical, and mental training. Howe (2005) emphasizes that channeling fear, anger, and anxiety through realistic training based on values is a key to controlling aggression. Boe and Ingdahl (2017) highlight the fine line between necessary aggression and excessive force, warning that deviation from ethical values can lead to dehumanization. Historical military examples, such as the My Lai massacre in Vietnam (Grossman, 2009) and abuses by British soldiers in Iraq (Brown, 2016), illustrate the dangers of uncontrolled aggression. Proper education, as seen in the Norwegian Army’s aggression control training, has yielded positive results (Boe and Ingdahl, 2017). During missions, focus should remain on techniques and tactics necessary to accomplish objectives (Boe, 2003; McCoy, 2007).

### 1.5 Prison riots and the need for a well-functioning combat mindset

Reiner (1997, p. 997) defined policing in its broadest sense as ‘the whole craft of governing a social order’, while Bayley (1995) defined it as “the activity of institutions authorized by the state to maintain internal order by enforcing the law, preventing crime, and providing services.” In the NCS a specific part of this is to deal with disturbances and prison riots. When defining riots and disturbances, it is important to distinguish between individual-level and collective-level disorder (Useem and Reisig, 1999). Individual-level disorder can involve a single prisoner using violence as a form of protest or engaging in a cell riot, where an inmate causes severe material damage or self-harm within their cell. In such cases, riot squads may be deployed to regain control, prevent self-harm, limit material damage, and contain the disorder before it spreads to other inmates, potentially escalating into a collective riot (Sørensen and Boe, 2026). Jore (2016, p. 169) defines “security” as “the perceived or actual ability to prepare for, adapt to, withstand, and recover from dangers and crises caused by people’s deliberate, intentional, and malicious acts such as terrorism, sabotage, organized crime, or hacking.”

Larger disturbances at a collective level are often classified as prison riots. Useem and Kimball (1989, p. 4) define a prison riot as occurring “when the authorities lose control of a significant number of prisoners, in a significant area of the prison, for a significant amount of time.” However, defining a riot so broadly overlaps with the broader category of disturbances. While acknowledging Useem and Kimball’s definition from 1989, this paper refines it by emphasizing that in a riot, aggression is not only present among different inmate groups but is also directed toward prison officials who govern the facility.

A study by Boe et al. (2023) found that reducing threats and assaults on prison officers in Norway requires several key measures: increasing staffing, ensuring follow-up from managers, enhancing managerial competence, providing general follow-up after incidents for both officers and operational managers, and allowing sufficient time to perform duties effectively This study also emphasized the importance of training exercises focused on prison-related challenges, aligning with the need to develop a well-functioning combat mindset for prison officers.

### 1.6 Factors affecting a well-functioning combat mindset

The Norwegian prison system is based on dynamic security, meaning prison officers interact closely with inmates to maintain safety and order (Sørensen et al., 2024). As a result, officers are frequently exposed to inmate frustration and potential violence. For particularly violent inmates held in long-term confinement, recognizing early signs of aggression and adjusting responses accordingly is crucial in preventing violence (Sørensen et al., 2024). Despite a declining number of inmates (Viggen, 2023), threats and assaults against prison officers in Norway have increased in recent years (Sygit-Kowalkowska et al., forthcoming).

Prison officers at the UCNCS are trained to handle riots (Kriminalomsorgens høgskole og utdanningssenter KRUS, 2025). However, it may seem that current training, which uses markers and tennis balls, lacks the severity of real-life situations. Developing a well-functioning combat mindset requires training in making quick decisions under uncertainty, as stress can threaten wellbeing and test coping abilities (Weiten, 2007). Henriksen and Kruke (2020) found that in armed confrontations, police officers rely on both experience and situational cues, combining analytical and intuitive decision-making. Stressful conditions and short distances often shape these decisions. Notably, Henriksen and Kruke (2020) reported that Norwegian police officers tend to withhold fire until threats escalate into actual attacks, placing themselves at imminent risk.

To enhance a well-functioning combat-mindset one part is to train and practice making quick decisions under great degree of uncertainty. As stated by Wes Doss, an instructor with over 30 years of military and law enforcement experience: *Greater than any other calling, the life of the warrior requires mental skills in combination with physical or mechanical skills. Yet, mental training is an area which has been long neglected in the fields of conflict management and force application* (2007, p. 65). These mental skill can be learned, but if the learned skill is just technical or physical they are of little value. Effective stress management in riot situations is crucial for maintaining control (Yanilov and Boe, 2020).

Situational awareness is essential for responding to threats effectively. Endsley (1995) emphasizes that continuously observing and analyzing surroundings allows for appropriate and proportional use of force. This skill is critical for prison officers managing high-stress situations like prison riots, where a well-functioning combat mindset is necessary for maintaining control.

## 2 Materials and methods

This article is grounded in an autoethnographic account of one of the author’s experiences. Autoethnography is a qualitative research method where the researcher uses personal experiences as primary data, blending ethnography with autobiographical elements (Muncey, 2010). It contextualizes personal reflections within broader cultural, social, and political frameworks. Midtlyng and Leseth (2024) explore the insider perspective of former prison officers researching their profession, aligning with the personal narrative style common in autoethnography. This approach makes research accessible and engaging, though it faces criticism as a method (Delamont, 2007; Tolich, 2010). Delamont (2007) criticizes autoethnography for its tendency toward self-indulgence and lack of sociological rigor, arguing that personal narratives often fail to connect individual experience to broader theoretical or social contexts. In this paper we have a critical reflection on the researcher’s experiences and how these relate to larger societal structures and phenomena. It is the combination of theory and experience in autoethnography where it integrates theoretical analysis with personal stories to illuminate how individual experiences can provide unique insights into larger cultural and social contexts.

Similarly, Tolich (2010) emphasizes the ethical risks inherent in autoethnographic work, particularly the exposure of others without consent and insufficient ethical oversight. In this study the autoethnographic description is from one of the authors that has fully insight into his exposure since he has written the autoethnographic description and is fully informed of the analysis in this paper.

In January 2024, the first and third author discussed critical prison incidents that had left a strong impression on them. This led to the decision to write an article based on the third author’s autoethnographic account of the prison riot at Oslo Prison on New Year’s Eve 2000, in which he served in the riot squad. The third author produced a chronological narrative of the event and his reflections on their involvement, which he shared with the first and second authors that analyzed the account by applying the theoretical framework of combat mindset, which became the article’s central concept.

Autoethnography combines theory and lived experience, offering deep insight into identity, culture, and social systems. It is especially useful for examining rare, high-stress events like prison riots, where firsthand accounts provide detail beyond traditional methods. In Norway, such riots are rare, making experienced officers’ valuable elite informants (Jacobsen, 2022).

### 2.1 Methodological critical reflection

While recent riots in Norwegian prisons have been smaller in scale, the Oslo prison riot on New Year’s Eve 2000 was a full-fledged event. When asked about it, the third author immediately responded, “I remember the riot as it was yesterday.” Reconstructing events after 24 years is challenging, though extreme situations are often vividly recalled. The third author retrospective account—from duty start to return home—may involve biases in relation to inaccuracies of recall, distortion, post-rationalization, blurring immediate reactions with later reflections, given the time that has passed. The autoethnographic description also may lack historical sensitivity (Tjora and Willis, 2006), and, of course, the fact that the third writer experienced an episode of substantial stress and possible trauma, even though he did not express such personal traumas. Still, such narratives offer valuable insights when no real-time records exist (Sosniak, 2006).

The analysis was primarily conducted by the first and second author, who did not participate in the riot. They based their analysis solely on the third author’s autoethnographic account, allowing all three authors to create a common understanding of the riot situation leading to a deeper insight using the concept of combat mindset as the central theoretical lens.

Translating from Norwegian to English in this autoethnographic study presented challenges (Patton, 2014), especially in balancing fact and interpretation and ensuring textual validity (Czarniawska, 2004). To maintain accuracy and authenticity, early drafts included interview excerpts in both Norwegian and English, preserving the original voice and intent.

## 3 Results

To explore our two research questions, we analyzed the third author autoethnographic account of a full-scale riot. This section presents a linear narrative in three parts, linking the empirical data to the concept of a well-functioning combat mindset and the need for realistic training for prison officers.

### 3.1 Research question 1: how does a prison officer’s experience during a riot align with the concept of a well-functioning combat mindset?

As will become evident from the description, this was a life-threatening situation that caused extreme stress, leading the third author to question his hesitation in using his baton. In the next section, we will explore this issue further to gain insights into research question 1: How does a prison officer’s experience during a riot align with the concept of a well-functioning combat mindset?

### 3.2 Combat mindset: turning on the switch in the head

*On 31.12.2000 I was 27 years old and relatively newly qualified as a prison officer. On this day, I was on 07–19 duty on ward D-1 and was hoping for a quiet shift, planned a New Year’s Eve party later in the evening and was eager to get home. At around 1830, we received a message from the control room that there was a disturbance in department A-3. Initially, we were told to stay calm in the ward. Shortly afterwards, the alarm went on. A lockdown was ordered, and we were told to meet outside the control room. The operations officer informed the attending officers that several cans of alcoholic beverages had been found earlier in the afternoon in the communal area of ward A-3 and that several inmates were intoxicated. It was therefore decided to lock them up before the scheduled time, but the inmates were aggressive and refused to comply with the order. The chief officer had attempted to negotiate with the inmates, but after a short time he was told to leave the unit by saying it would be dangerous for him to stay there. After the joint briefing, the chief officer selected 11 officers to carry out an operation in the department. I was one of them. We were ordered down to the basement to put on protective equipment—i.e. overalls, gloves, shields, shin guards, batons and helmets. We were then ordered up to the third floor—outside the door to department A-3. The plan was to form a barrier line across the ward, and then move in this formation until we had pushed the inmates toward the opposite end of the ward—to arrest everyone there. The door was unlocked, and we quickly formed a barrier line. About 10 of the inmates asked to be locked in their cells*.

Intoxicated inmates that are aggressive and that explicit telling the chief officer that it is dangerous for him to be in the wing is a situation where officers understand that the need for physical intervention is needed and is necessary to turn on a combat mindset. Howe (2005) notes that channeling fear, anger, and anxiety over many hours of relevant realistic physical and mental training that is based on values is beneficial to adjust to and to use the correct level of aggression. Hassmén et al. (2003) state that well-developed psychological skills are a prerequisite for being able to perform at a high level, regardless of the type of performance.

In this excerpt we can estimate that several factors, such as reverse conditioning, turning on the switch in one’s head, a dismissed dialogue and that putting on your equipment, may have affected the prison officer during the riot situation. Your performance will in turn be affected by various factors such as your activity and level of achievement (Humara, 1999; Martens et al., 1990). The activity and achievement levels can be changed through physical training, visualization, breathing, concentration, and focusing practice.

Conditioning is a model for how much of human emotion is learned and is useful for understanding how stress is created in humans (Weiten, 2007). When the prison officer experienced a threatening situation, he can condition himself to become stressed. Perceive a threat, the inmates starting a riot (the unconditioned stimuli) may elicits anxiety (the unconditioned response). The orders to get ready (known as a conditioned stimuli) as in meeting outside the control room because of expecting a threat (an unconditioned stimuli) leads to arousal and/or anxiety (an unconditioned response). Next time the orders to get ready (a conditioned stimuli) may then lead to arousal and/or anxiety (a conditioned response). Same with putting on equipment, this may lead to the same type of conditioned response with arousal and/or anxiety (Yanilov and Boe, 2020). Without training, this scenario repeats itself. Putting it simpler, you learn to be afraid when the orders are coming, or you have to put on your equipment. However, if you handle the challenge successfully, you instill confidence and desensitize yourself to anxiety. This process is called reversed conditioning. Police officers and soldiers train behavioral patterns through drills to benefit from this phenomenon. Reversed conditioning creates self-efficacy—your belief influences your ability to handle stressful situations (Bandura, 1997). It also means you learn to turn on the switch inside your head in a correct way, that is, applying a well-functioning combat mindset (Yanilov and Boe, 2020).

### 3.3 Combat mindset: stress management and decision making

The autoethnographic description continues: *The remaining 11 appeared very aggressive. They had prepared weapons consisting of table legs, boiling water, kettles, and other kitchen equipment. They ran toward the barricade line in an arc formation and threw tabletops, boiling water, hit us with clubs, threw burning curtains etc. at us. Eventually they also sprayed us with the fire hose. Several of the officers suffered minor injuries or were replaced because they found the situation so demanding that they were unable to carry out their assignment.*

*My place in the line was far to the right, and I only had two officers between me and the right wall with cell doors. The officers on the far right and left were tasked with locking the cell doors as we passed them. Because of the attacks from the inmates, I had my baton raised to strike all the time. I understood that the situation was dangerous, but I felt completely calm. I had no experience from a similar situation, nor had I ever used a baton before, even though we had carried out some relevant exercises during my officer training, I had no idea where on their body I should strike, or whether the force of the blow should be adapted to the situation. In other words, I was prepared to hit the head with full force if necessary. On a couple of occasions, the inmates got hold of my shield in the cordon line. They tried to deprive the officer of the shield but were unsuccessful. The lamps in the ceiling were torn down and smashed. Apart from the light from burning curtains and bookshelves, it was dark in the wing. We stood in several centimeters of water and debris, and I wondered whether water in the electrical system could create a risk of dangerous shocks. At one point, one of the inmates shouted, ‘get the gun’. There had been rumors in recent weeks that a gun had been smuggled into the prison, so the shout drew my attention. I remember thinking that if they have a gun, we are screwed. We did not have bulletproof vests, but no gun appeared, and the action continued*.

From this partial excerpt of the riot, we see that the perception of escalation, a false sense of confidence, being unconscious incompetent and experiencing parallel stressors all were factors contributing to the prison officer’s experience of the riot. Parallel stressors are stressors that occur at the same time, leading to an increased level of stress (Boe, 2015).

Perceived competence within clearly defined domains or activities has been found to be the main factor in both self-perception and self-efficacy (Eccles et al., 1998). Research shows that performance may be affected by the degree of perceived anxiety (Humara, 1999; McKay et al., 1997; Woodman and Hardy, 2003). The more anxiety, the worse your performance. The more confidence, the better your performance (Siddle, 1995).

In mission-critical situations, susceptibility to psychological stressors can be a determining factor in whether one succeeds or fails (Wherry and Curran, 1966). Research has identified a variety of stressors that impairs problem-solving abilities, including auditory distractions (Broadbent, 1978), performance pressure (Baumeister, 1984), workload (Wickens, 1979), anticipated physical threat (Wachtel, 1968), and dangerous environments (Burke, 1980; Hammerton and Tickner, 1969). These stressors have been shown to increase physiological arousal, leading to elevated heart rate, labored breathing, and tremors (Rachman, 1995). Under stress, individuals’ problem-solving capabilities decline (Yamamoto, 1984), and they exhibit more rigid task execution (Staw et al., 1981). Performance stress alone has been shown to triple the rate of errors in operational tasks (Villoldo and Tarno, 1984), and manual task completion time can double under high-stress conditions (Idzikowski and Baddeley, 1983). One of the most challenging aspects of stressful and uncertain situations is decision-making (von Schell, 1933). The challenge stems from information arriving at different times. People often hesitate, waiting for more data to reduce uncertainty—but this delay can hinder timely decisions in operational settings (Lipshitz, 1997). Sudden, uncontrollable, or unpredictable situations can trigger intense stress, closely tied to a person’s ability to regulate emotions (Boe, 2015). The autoethnographic account shows that the riot severely challenged the officer’s capacity to maintain a trained combat mindset.

The data show that even newly trained officers with riot training may feel uncertain under stress—such as not knowing where to strike with a baton during an attack. Although the officer remained calm, he hesitated and questioned his actions in the midst of the conflict. To develop an aggressive and functional combat mindset, officers must first recognize the situation as demanding and requiring such a mindset. This involves training both technically and physically, with a clear understanding of their tasks and how to carry them out (Yanilov and Boe, 2020). Realistic scenario-based training is also essential to reduce unfamiliarity with stress responses and improve decision-making in self-defense situations.

Another key aspect of riot preparation is understanding the legal guidelines for using a baton—specifically where and when to strike when an inmate poses a direct threat, such as attacking the front line and threatening the officers security. Uncertainty in such moments can cause hesitation, weakening what Siddle (1995) describes as the essential determination required for a combat mindset.

Garcia (1989) found that under stress, police officers often relied on the simplest baton techniques, despite training in more complex ones. Uncertainty about where to strike can undermine an officer’s focus, concentration, and self-control—key elements of an effective combat mindset. Adding to this challenge, baton skills are difficult to acquire and even harder to retain (Waddington and Wright, 2008).

There are techniques to build a well-functioning combat mindset. According to Grossman (2009) there are two learning mechanisms (psychological techniques) called classical and operant conditioning that helps soldiers to operate even under extreme stress in combat. The first of the two learning mechanisms are according to Grossman, classical conditioning, is a form of personal learning connecting stimulus with a response. The second learning mechanism, operant conditioning, means giving a person a reward to reinforce an action that is a form of positive reinforcement (Weiten, 2007). In a prison riot, serious violent attacks or other prison disturbances involving significant stress will challenge one’s stress tolerance, but also past experiences and the exponent of realistic training before entering a fight. These experiences and training will in addition to the similarity to the situation affect strongly a person’s ability to respond and function, but also to have a withstanding capacity and functional combat mindset. The main thing to develop a combat mindset is to practice relevant exercise regularly. It will then be more convenient to deal with stress in an acute stress situation if the person has trained different drills that comply to different stress situations. Boe et al. (2011, 2012) argue to drill the tasks a person is going to achieve and that counteract a possible paralysis in a stressful situation. The aim of the drill is to counteract a possible paralysis that can appear due to stress (Boe, 2011; Boe et al., 2020). By showing the correct response to the stimulus, this will reinforce the belief in the decided action, and the nerve connections will become faster by repeating it until automation of an action (drill) is obtained. Simply put, it is all about stimulus—response (Boe and Ingdahl, 2017).

Moldjord and Holen (2005) argue that drills help build procedural memory—memory for actions and routines. Riot squads entering prison disturbances follow detailed, structured procedures for movement and response. While operations are order-based, officers must also adapt to unfolding events. For example, an officer may step out of formation to strike an aggressive inmate if the situation demands it. Sde-Or Lichtenfeld and Yanilov (2001) emphasize that accurately identifying and understanding a situation is key to developing a combat mindset. A clear perception of a threat can trigger faster responses. For prison officers, recognizing a “worst-case scenario” allows them to activate an offensive mindset more quickly—provided they have mentally prepared for such situations in advance. Physical training is resource-intensive, so mental training—such as visualizing the mission, as described by Doss (2007)—can be a useful alternative. While it cannot replace realistic physical training for practical tasks, mental training helps shift negative thoughts to positive ones, which is vital for building an effective combat mindset. Techniques like positive affirmations (e.g., “I can handle it”) can also reduce anxiety, self-doubt, and worry. According to Limbert (2004) during the Falklands War in 1980, the majority of British military used positive coping strategies as their primary strategy.

### 3.4 Combat mindset: situational awareness and training methods

The autoethnographic description continues: *At the end of the corridor - behind the screaming and rabid inmates - there were large, barred windows. Outside, it was completely dark, and we could see burning objects being thrown out of cell windows in the other wards. There was a lot of shouting and screaming and we could hear the sound of crushing from several cells as well. THE WHOLE BUILDING WAS SHAKING. My impression was that the riot had spread to other wards - although it later turned out to be just a handful of individual cell shattering.*

*A few minutes later, the dark and smoky premises were illuminated by flashing blue lights from outside. We realized that the police were on their way. We therefore stopped our advance and stood in the barricade line—about 20 m into the cell block. Shortly afterwards, we heard powerful dog barks and loud POLICE! MAKE WAY!’ command. We opened the barrier and several police officers with dogs shouted, ‘GET DOWN!’ This was a command that all inmates immediately complied with. Some got down on their knees and elbows—presumably to avoid lying down in the water. One of the police officers pacified them by jumping on their lower back with the effect that they were immediately left fully stretched out. A female police officer handed out handcuffs from a large cardboard box—and in cooperation between police and prison officers, all inmates were arrested and handcuffed within a few minutes. Shortly afterwards, I was ordered directly to prisoner transportation and drove inmates who had participated in the riot to several prisons in the eastern part of the country. I got home from work at 0830—just over 25 h after starting my shift at 0700*.

*Even though I felt calm I later realized that my perception of time was very distorted since the operation went very slowly—an estimated 2 h, while a report later showed that the operation was completed in less than an hour*.

This excerpt shows that the riot was perceived very differently from routine or expected events, highlighting the need for more realistic training methods and scenarios. The officer experienced extreme stress, which affected situational awareness and created a sense of time distortion. Enhancing situational awareness and rapid decision-making—especially regarding use of force—is essential for handling such incidents effectively (Yanilov and Boe, 2020).

A significant increase in heart rate caused by hormone-induced activation of the nervous system can be caused by being scared, stress, fear and uncertainty. A person’s ability to think clearly and process information drops when the heart beats rise over 145 beats per minute (Grossman, 2009). Siddle (1995) argues that using “tactical breathing” it is realistic to be between 115 and 145 beats per minute where brain and body functions are more optional together in this zone. Asken et al. (2010) also argues for practicing “tactical breathing” as a part of a well-functioning combat mindset since it can lower the heart rate to a level a person can function optimally. According to Grossman and Christensen (2008) this is the zone optimal for combat performance and survival since it gives a person the ability to control his body and remain well in this zone by taking control of the breathing when getting into a stress situation. Boe (2006) shows how a technique referred to as Adrenaline Stress Conditioning (ASC), combining practical tactical breathing and training with adrenaline and stress hormones in the body, work well despite the hormone-induced stress rises above 145 beats per minute. The main issue is in a stressful situation to practice remaining between 115 and 145 heartbeats per minute, and if the situation escalates and the heart rate increases above that the goal is to function effectively with large amounts of adrenaline and hormones (Boe, 2003). Siddle (1995) shows us how a person loses the ability to think rationally, but also the ability to remember a situation and sequence under extreme cognitive load. Critical incident amnesia (Grossman and Christensen, 2008; Grossman and Siddle, 2001) happens when the heart rate rises above 175 beats per minute. It is possible to overcome such a state of mind by mentally going through possible scenarios, to take advantage of breathing techniques, and take control of the body even in this extreme situation.

Under stress, your coordination, cognition, and memory may be impaired. You may suffer tunnel vision, loss of hearing (“auditory exclusion”), and a distorted perception of time and space (Klinger, 2004). Also, Syrigou et al. (2025) reported that stressful environments can significantly alter how people experience time, causing it to either speed up or slow down, and this may explain the perception of time distortion for a prison officer engaged in a riot.

### 3.5 Practicing a well-functioning combat mindset

When practicing prison officers combat mindset, it is beneficial to discuss sequences of training with stressors and problems to be aware of their performance, behavior and coping skills in relation to their capacity for action in these situations. The reflection phase incorporates a common situational awareness in all team-members toward the interaction processes during the training of cooperating to solve a mission. According to Torgersen (2018) reflection is beneficial linked to self-efficacy of sequences during training that incorporates sudden, unscheduled, and unexpected events. Torgersen (2015, 2018) argues that reflection phases should be linked closely to the well-known concept of “train as you fight” originating from the US Army General William F. DePuy (Rietjens et al., 2013). The different phases of reflection should be linked to action-oriented training activities closely related to the field where they operate. In some contexts, it is maybe best to implement reflection in training as independent sequences, especially reflection of awareness, dilemmas and ethically charged situations. Practical training should also be included since it is necessary to practice cognitive meta reflections under stress and time pressure. Training reflection systematically in relation to operational preparedness is a necessity to enhance a progressive combat mindset.

Another aspect that can influence and possibly interfere with an offensive combat mindset is that an officer’s main work tasks are about care and rehabilitation. Switching mentally from a strong tradition with care and humanity as described by Pratt (2008a) as part of Nordic Exceptionalism, can be both cognitive and emotional demanding to shift to a combat mindset when the situation demands it. Shifting from a combat mindset during a crisis to a care-oriented role is also demanding as has been postulated by Krulak (1999). However, awareness that care responsibilities later will follow critical incidents may encourage prison officers to incorporate a humane perspective even during severe incidents.

Sde-Or Lichtenfeld and Yanilov (2001) claims that the ability to recognize and identify when an emerging situation has the need of a combat mindset is central since identify triggers leads to a faster establishment of a constructive combat mindset. This deals with whether the NCS values the need for an aggressiveness that is part of a combat mindset or not. Without valuing aggression as a necessity that are needed in prison riots but also acknowledging that prison officer needs a well-functioning combat mindset in specific situations even though it is consequently different with the most valued statements in the Norwegian White Paper (Justis-og beredskapsdepartementet, 2008). However, learning to control aggression becomes an important element of functioning in a more normal environment (Brown, 2015).

### 3.6 Research question 2: is there a need for more stressful training scenarios for prison officers to foster this mindset?

There is a common understanding that aggression is considered to be something stemming from wanting to create damage to another organism (Baron, 1977; Berkowitz, 1989; Buss, 1961; Dollard et al., 1939; Zillmann, 1979). Murray (2006) refers to two ways to develop and train the necessary degree of aggression; skills and coping with stress. For a soldier to have control of his or her exercising of aggression, in principle four assumptions that must be present. These are: self-knowledge and self-control, the opportunity to let off steam, social support and conversation with someone you have confidence (Moldjord and Holen, 2005).

At the UCNCS, prison officers are trained to deal with a riot and stress where figurants are acting as inmates, shouting, playing loud music and throwing tennis balls on their shields to simulate aggression and noise.

In the autoethnographic description from the riot in Oslo prison describe the situation with a dark wing, flames, and smoke from burning curtains, boiled water is being thrown at the officers, but also frying pan are being thrown at the officers’ legs so they have to jump to get clear of these flying objects at the same time they are traying to hold the line in the riot squad. It was extremely noisy, and the prison building was shaking because of the smashing of the inventory. To build up a combat mindset to deal with such riot UCNCS must train with similar circumstances. The last training sessions in dealing with a riot can be advantageous that the student have the technical training and know how and when to hit with their baton, while they at the same time will be exposed with similar stress moments as it was in Oslo prison in 2000. Making loud noise, turning off the light to get a dark wing, using smoke-machines to get the impression that something is on fire and throwing objects on the officers’ legs create a realistic event that we have learned from this autoethnographic description that students in advantage must be trained to deal with. Having said that, it is also equally important to develop a culture of controlled aggression by channeling fear, anger and anxiety through many hours of realistic, relevant physical and mental training that is based on values (Boe and Ingdahl, 2017). By values, we mean the will to do the right thing based on correct values in order to install correct aggression and not a misdirected one involving too much or uncontrolled aggression (Boe et al., 2020).

One question that can be raised is why should prison officer students train on something that seldom occurs and that very few officers must deal with? We have shown that NCS have full-fledge riots where they need assistance from the police and disturbances that are demanding and can evolve into a riot.

Prisons in Norway experiences cell riots that regularly have the same mechanisms as a collective riot with inmates smashing inventory, lightning their cell and scream loudly. If prison officers train on more severe situations like a full-fledge riot these cell riots becomes probably less demanding since they have trained in more demanding situations. When a riot is ongoing or the disturbances are so severe that the officers are thinking that now we will have a riot, they will be more assured that they as a group can deal with a riot, since they have dealt with a realistic riot under training. As a prison officer it is reassuring to know that he or she is trained to deal with a riot and this is building a well-functioning combat mindset in line with what Siddle (1995) and Grossman (2009) describe as courage, determination, mental stamina and the necessary aggression to go into a fight mode and deal with a riot in an appropriate way.

## 4 Conclusion

Our aim in this article was to explore a prison riot in Oslo, Norway, with a focus on two key research questions: RQ1: How does a prison officer’s experience during a riot align with the concept of a well-functioning combat mindset? RQ2: Is there a need for more stressful training scenarios for prison officers to foster this mindset? Using an autoethnographic case of a prison riot described by the third author, we found the following answers to our two research questions. Our findings indicate that the officer’s experience largely supports the combat mindset concept, answering our first research question. Additionally, the study concludes that substantial training is necessary to develop a beneficial aggression level and thus helping officers effectively manage high-stress situations, addressing the second research question.

A report from Rambøll Management Consulting (2023) show also that prison officers work in Norway have severe personal consequences for their health. To be better prepared for a riot they must be given more insight into a well-functioning combat mindset.

These findings give us insight into the severe stress that an officer has experienced in a full fledge riot and thereby give us the opportunity to understand the major stress in this situation and the importance of a developed combat mindset. This combat mindset must have been enhanced during training where the officer is well-educated in what he can use of power and force in a riot where the officer must rely on his operational skill since the time to reflect about what to do is nearly impossible in a full stress situation.

Based upon our research, we therefore recommend the NCS implement and training even more realistic scenarios with severe stress in order to cope with possible prison riots. Failing to install a well-functioning combat mindset in prison officers may lead to what is known as an over-determined reaction to the riot. An over-determined reaction means that a person exhibits a stronger reaction to an event than what one would normally assume (Holen, 2005), potentially causing long-time psychological consequences for the person experiencing this.

This study is in line with previous research on prison officers in single-cell riots (Sørensen and Boe, 2026), police officers in the 2011 terror attack (Boe et al., 2020) and Henriksen and Kruke’s (2020) report that Norwegian police often delay firing until threats become actual attacks yet hesitation can place them in imminent danger. For soldiers (Boe and Ingdahl, 2017) highlights the importance of a well-functioning combat mindset for balanced responses under extreme stress. We therefore recommend further case studies to deepen understanding of security professions (soldiers, police officers and prison officers etc.) who are dealing with people who have an intention to harm others, so they can improve learning and preparedness in frontline operations.

### 4.1 Limitations

The third author may identify closely with the organization, potentially favoring its values over personal views. This could have influenced the data to align more with the organizational perspective or the author’s own interests. However, there is no clear sign of deliberate bias. Despite possible unconscious influence, his retrospective account provides valuable insight into the Oslo Prison riot on New Year’s Eve 2000.

## Data Availability

The raw data supporting the conclusions of this article will be made available by the authors without undue reservation.
